# What is the role of attitudinal barriers on cervical cancer screening non-attendance? Findings from a cross-sectional study with migrant women in Portugal

**DOI:** 10.1186/s12905-023-02198-2

**Published:** 2023-02-09

**Authors:** Patrícia Marques, Mariana Geraldes, Ana Gama, Bruno Heleno, Sónia Dias

**Affiliations:** 1 NOVA National School of Public Health, Public Health Research Centre, Comprehensive Health Research Center, CHRC, NOVA University Lisbon, Avenida Padre Cruz, 1600-560 Lisbon, Portugal; 2 NOVA Nacional School of Public Health, Public Health Research Centre, NOVA University Lisbon, 1600-560 Lisbon, Portugal; 3 CHRC, NOVA Medical School, NOVA University Lisbon, 1169-056 Lisbon, Portugal

**Keywords:** Early detection of cancer, Emigrants and immigrants, Reproductive health, Uterine cervical neoplasms, Women’s health

## Abstract

**Background:**

Cervical cancer is a common disease which can be effectively and timely detected by cervical cancer screening. However, access to cervical cancer screening is unequal, and it is known that migrant women have a lower attendance to cervical cancer screening. These inequalities are associated with several factors, including attitudes and beliefs of the women regarding screening practices, which prevents them from participating. This study aims to explore the attitudinal barriers to cervical cancer screening among migrant women in Portugal.

**Methods:**

A web-based cross-sectional survey was conducted with 1100 migrant women residing in Portugal. Women were recruited through social media platforms. The survey included items on socioeconomic characteristics, cervical cancer screening history and an 11-item attitudinal questionnaire to assess attitudinal barriers. Logistic regression models were used for statistical analysis.

**Results:**

The attitudinal barriers to CCS most often reported by participants were fear of the test result (25.3%), worry about seeing a male health professional (23.8%), perceiving the test as painful (23.1%), embarrassment (18.5%), difficulties scheduling the test (14.3%), and having a negative experience in screening (12.4%). Low perceived need in absence of symptoms and lack of motivation to be screened were reported by less than 5% of the women. However, the results suggest that most of the attitudinal barriers with higher agreement percentage have no association with cervical cancer screening attendance. Among all the attitudinal barriers, low perceived need of screening and lack of motivation were associated with CCS non-attendance.

**Conclusions:**

Based on the findings, out of all the factors analyzed, low perceived need of screening and lack of motivation are the most relevant factors associated with non-attendance among migrants in Portugal. Promoting health literacy and empowering women with knowledge about benefits of screening may help overcoming these barriers. Therefore, this study provides a foundation for stakeholders on which areas should be prioritized when developing strategies aiming to reduced cervical cancer screening non-attendance among migrant women.

## Introduction

Cervical cancer is a common and preventable disease. HPV infection of the cervix may originate pre-cancerous lesions that, if left untreated, can develop into cancer [1–3]. Cervical cancer screening (CCS) is a health intervention that helps to identify HPV infection and treat pre-cancerous lesions to avoid cervical cancer development [4, 5]. Evidence suggests that population-based CCS programs are effective to reduce mortality from cervical cancer [4–6]. However, even in countries with organized cancer screening inequities persist, namely low screening participation of migrant women [5–7] In Portugal, CCS is performed opportunistically and within the population-based program. The population-based CCS program is offered free of charge to all women between 25 and 65 years old, who are registered in a healthcare unit (including documented migrants) [8, 9]. Women who underwent hysterectomy or had previous cervical cancer diagnosis are excluded from the program [10]. CCS is performed every 3 or 5 years and the testing method is cytology that can be complemented with HPV testing (depending on the geographic location of the healthcare unit), usually performed by a general practitioner (GP) [8, 9].

Portugal has been traditionally a host country for migrants from Portuguese-speaking African Countries and Brazil, and more recently from Eastern Europe, China, and South Asia [11, 12]. In these countries, the prevalence of high-risk HPV infection, and the incidence and mortality of cervical cancer are high [13]. According to official data from the Portuguese Immigration and Borders Service (SEF), in 2021 there were 698.887 foreign residents in Portugal, of which 46.2% were women [14]. Results from the Portuguese National Health Survey (2014 NHS) suggest a higher rate of CCS non-attendance among women with a foreign country of birth (16.7% vs 12.8% among natives) [15].

Factors associated with CCS non-attendance among migrant women have been explored. Several studies have consistently reporting lack of knowledge about screening, language difficulties, migration-related factors and difficulties in accessing healthcare services as barriers to CCS [6, 7, 16–20]. Women may avoid CCS as they may feel discriminated because of their migration status, language skills, cultural beliefs, or even physical traits (e.g. high BMI) [7, 21]. Other factors such as attitudes, beliefs and perspectives on CCS, namely perceived risk of cervical cancer, negative past experiences, having other life priorities or having to see a male doctor, may have additional effects on CCS attendance. Nevertheless, these factors have been explored mainly by qualitative studies [22–27]. To develop effective strategies to increase CCS participation it is key to quantify the most prevalent barriers to CCS and measure their association with non-attendance. To the author’s knowledge, there is only one study aiming to explore the association between attitudinal barriers and CCS non-attendance. This study was developed by Marlow et al. [16] and consisted of a survey conducted in England with women of different ethnic groups (i.e. from Indian, Pakistani, Bangladeshi, Caribbean, African and White British backgrounds), that explored sociodemographic and attitudinal correlates of CCS non-attendance.

In summary, this study showed that a lower CCS attendance was associated with a low perceived risk of cervical cancer and the belief that screening is unnecessary in the absence of symptoms, with significant ethnic differences regarding most of the attitudinal barriers assessed. Other quantitative studies are needed to further understand the role of attitudinal barriers on CCS non-attendance in other countries where evidence is scarce, as Portugal, to develop strategies targeting these women and their specific needs.

This study aimed to assess attitudinal barriers to CCS and examine its association with CCS non-attendance among migrant women in Portugal.

## Methods

### Study design and sample

This cross-sectional study consisted of a web-based survey. The inclusion criteria were being a woman, migrant (not born in Portugal), currently living in Portugal and aged 20 years or older. For this study, a migrant is defined as “any person who is moving or has moved across an international border or within a State away from his/her country of origin” [28].

Participants’ recruitment was made through online campaigns in social media platforms, including informal online migrant groups and official pages of organizations that support migrant communities in Portugal. After asking permission to the moderators of the social media pages and groups, a link to the survey was disseminated. Visitors interested in taking part of the survey could access the survey page by clicking in the link provided.

### Data collection

The survey was conducted between February and July of 2021 using Google Forms. A Portuguese and an English version of the survey was provided. A pre-test of the survey was conducted with 10 voluntary participants. This ensured that the survey platform was working properly, and the questions were clear. When accessing the survey’s link, participants were directed to an informative page with the description of the study, the contacts of the research team and ethical considerations including anonymity and confidentiality. Participation was voluntary, and participants were free to skip questions or leave the survey at any moment. Only after participants filled the informed consent form, they were able to access the survey questions. No incentives were offered to the participants in exchange to their participation in the study. A total of n = 1165 women accessed the link of the survey and of these n = 8 women declined to participate.

The survey included items on participants socioeconomic characteristics (age, continent of birth, education, employment status, marital status, having children), migration-related characteristics (current migration situation, length of stay in Portugal), healthcare-related characteristics (lifetime GP appointment in Portugal, last gynecological appointment, having a family doctor in Portugal, HPV vaccine), as well as items on CCS history and attitudinal barriers experienced by the participants.

To explore CCS history of the participants, women were asked “Before participating in this study, have you ever heard of cervical cancer screening (Pap smear)?” and “Have you ever participated in cervical cancer screening (Pap smear)?”, with Yes/No response options. Women who reported to have been screened before were asked “When was the last time you had cervical cancer screening (Pap smear)?” with four options of answer: “I did it last year”, “I did it between 1 and 5 years ago”, “I did it over 5 years ago”, and “I don’t know”. A new variable “Having ever been screened” was created with the response options “No”, “Yes, ≤ 5 years ago” and “Yes, > 5 years ago”.

Attitudinal barriers were explored using 11 attitudinal statements, based on Marlow et al. [16] work. These attitudinal statements were related to four themes: perceived need for screening, fear of cancer, concerns about the test and practical considerations. Women were asked to position themselves in relation to each statement using a 5-point Likert scale (strongly disagree, disagree, neutral, agree, strongly agree). For the statistical analysis, these response options were dichotomized into “disagree” (including strongly disagree, disagree, and neutral) and “agree” (included strongly agree, and agree).

### Data analysis

Data collected was stored in Google Forms platform and transferred to an Excel file. Statistical analysis was conducted with IBM SPSS – 27 version IBM SPSS Statistics for Windows (Armonk, NY: IBM Corp.).

Data was screened for duplicates by comparison of three variables—country of birth, date of birth and employment status; no duplicate responses from individual participants were found. Women were excluded from data analysis if they did not provide information on their continent of birth or CCS participation. Women who did not participate in CCS in their lifetime or whose last CCS was over 5 years ago were considered non-attenders, as 5 years is the recommended upper limit of screening intervals in Portugal [9].

A descriptive analysis of the sample characteristics and the attitudinal barriers to CCS was conducted. Bivariable and multivariable logistic regression models were performed to explore the associations between the attitudinal barriers and CCS non-attendance. Further analyses using Chi square tests were performed to explore differences in endorsing attitudinal statements associated with CCS non-attendance, according to sociodemographic, migration-related, and healthcare-related characteristics. Exact Fisher’s test was used when appropriate.

## Results

### Characteristics of the participants

A total of 1157 migrant women completed the survey; 57 were excluded for missing information on country of birth or CCS attendance. Hence, the study sample included n = 1100 migrant women. The characteristics of the participants are presented in Table 1. In brief, most women were ≤ 45 years old (61%). About 41% were originated from Central and South America (mainly Brazil) and 39.5% from Europe, with a smaller percentage of women from Africa (7.1%), Asia (5.9%) and North America and Oceania (6.6%). It was observed that 73.2% had a university degree and 24.7% high school education, 48.7% were non-employed, 68.9% were married or were living with a partner, and 56.9% had children. Out of all the participants, 12.3% were undocumented and 19.5% were living in Portugal for > 10 years.

**Table 1:** Socioeconomic, migration-related, health-related, and CCS related characteristics of the study participants

	Study participants (n = 1100)	
	n	%
*Socioeconomic characteristics*		
Age (n = 1063)		
< 45 years	651	61.2
≥ 45 years	412	38.8
Continent of birth (n = 1100)		
Europe	435	39.6
Africa	78	7.1
Asia	65	5.9
North America and Oceania	73	6.6
Central and South America	449	40.8
Education (n = 1096)		
Elementary or middle school (≤ 9 years of school)	23	2.1
High school (10–12 years of school)	271	24.7
Higher education (university)	803	73.2
Employment status (n = 1093)		
Employed	561	51.3
Non-employed	532	48.7
Marital status (n = 1097)		
Married/living with a partner	756	68.9
Single/separated/divorced/widow	341	31.1
Having children (n = 1097)		
Yes	624	56.9
No	473	43.1
*Migration-related characteristics*		
Current migration situation (n = 1093)		
Documented	959	87.7
Undocumented	134	12.3
Length of stay in Portugal (n = 1099)		
≤ 10 years	886	80.5
> 10 years	214	19.5
*Health-related characteristics*		
Lifetime GP appointment in Portugal (n = 1100)		
Yes	849	77.2
No	251	22.8
Last gynaecological appointment (n = 1100)		
< 5 years	806	73.3
≥ 5 years	294	26.7
Having a family doctor in Portugal (n = 1098)		
Yes	593	54.0
No	505	46.0
HPV vaccine (n = 1100)		
Yes	163	14.8
No	937	85.2
*CCS-related characteristics*		
Have you ever heard of CCS? (n = 1100)		
Yes	1053	95.7
No	47	4.3
Have you ever been screened? (n = 1100)		
Yes, ≤ 5 years ago	832	75.6
Yes, > 5 years ago	145	13.2
No	123	11.2

Most women stated having already had at least one GP appointment in Portugal (77.2%), even though only 54% had a family doctor attributed. Almost three quarters had their last gynecology appointment less than 5 years ago, while 24.4% had their last screening over 5 years ago or were never screened. Nearly all women have heard about CCS (95.7%) and 14.8% were vaccinated against HPV.

### Attitudinal barriers to CCS

Table 2 presents the 11 attitudinal barriers organized in four categories: “Perceived need for screening”, “Fear of Cancer”, “Concerns about the test” and “Practical considerations.

**Table 2:** Percentage of agreement with the attitudinal barriers, and logistic regression models (crude and adjusted) of the attitudinal barriers and CCS non-attendance among migrant women

	Agreement with the items	Crude model		Adjusted model^1^	
	n (%)	OR (CI95%)	*p*	OR (CI95%)	*p*
*Perceived need for screening*					
I am not at risk of cervical cancer, so I don’t need a smear test	70 (6.6)	1.039 (0.590–1.829)	0.895	1.223 (0.606–2.471)	0.574
I’m not sexually active so I don’t need to go for a smear test	23 (2.2)	6.315 (2.645–15.076)	< 0.001	2.919 (1.024–8.316)	0.045
I do not need a smear test if I do not have any symptoms	19 (1.8)	4.531 (1.802–11.391)	0.001	5.521 (1.731–17.613)	0.004
*Fear of Cancer*					
I’m scared of what a smear test might find	269 (25.3)	0.932 (0.671–1.295)	0.676	0.870 (0.583–1.298)	0.484
I don’t want to know if I have cancer	25 (2.4)	3.674 (1.654–8.160)	0.001	2.999 (1.135–7.920)	0.027
*Concerns about the test*					
Smear tests are embarrassing	198 (18.5)	0.951 (0.661–1.369)	0.787	0.654 (0.415–1.032)	0.068
Smear tests are painful	247 (23.1)	0.679 (0.475–0.970)	0.033	0.660 (0.430–1.013)	0.057
I’ve had a bad experience of a smear test in the past	132 (12.4)	0.506 (0.304–0.841)	0.009	0.391 (0.214–0.715)	0.002
I am worried I will have to see a male doctor or nurse	253 (23.8)	0.849 (0.603–1.194)	0.346	0.820 (0.540–1.246)	0.353
*Practical considerations*					
I intend to go for a smear test, but I don’t get around to it	39 (3.7)	2.572 (1.344–4.925)	0.004	2.943 (1.327–6.527)	0.008
It is difficult to get an appointment that fits with commitments	152 (14.3)	1.076 (0.723–1.601)	0.719	1.232 (0.759–2.000)	0.398

^1^Adjusted to age, continent of birth, education, employment status, marital status, having children, lifetime GP appointment in Portugal, last gynecological appointment, having a family doctor in Portugal, and HPV vaccine

Out of the 11 attitude items analyzed in the study, the most commonly endorsed were those under the categories “Fear of Cancer” and “Concerns about the test”: women agreed the most with the item “I’m scared of what the test might find” (25.3% of agreement), followed by “Smear tests are painful” and “I am worried I have to see a male doctor or nurse”, endorsed by over 23% respectively. The category “Perceived need for screening” was the one with the lowest overall agreement percentage (1.8–6.6%).

### Attitudinal barriers associated with CCS non-attendance

The results of the crude and adjusted logistic regression analyses of the attitudinal barriers associated with CCS non-attendance are detailed in Table 2.

Regarding the statements associated with perceived need for screening, women who endorsed the item “I’m not sexually active so I don’t need to go for a smear test” were most likely to be non-attenders in crude and adjusted model (aOR: 2.919; CI95%: 1.024–8.316). The same was observed for women who agreed with “I do not need a smear test if I do not have any symptoms” (aOR: 5.521; CI95%: 1.731–17.613).

In terms of the items associated with Fear of cancer, only the statement “I don’t want to know if I have cancer” was associated with screening non-attendance in crude and adjusted models (aOR: 2.999; CI95%: 1.135–7.920).

Considering concerns about the test, agreement with “Smear tests are painful” was associated with CCS non-attendance but only on the crude model (OR: 0.679; CI95%: 0.475–0.970). On the other hand, women who endorsed the item “I’ve had a bad experience of a smear test in the past” are less likely to be CCS non-attenders, both in crude and adjusted model (aOR: 0,391; CI95%: 0.214–0.715).

Finally, within practical considerations, only the statement “I intend to go for a smear test, but I don’t get around to it” was associated with CCS non-attendance in both crude and adjusted models (aOR: 2.943; CI95%: 1.327–6.527).

Further analyses explored the sociodemographic, migration-related, and healthcare-related characteristics associated with the statistically significant attitudinal barriers associated with CCS non-attendance (Table 3). The percentage of agreement with the attitudinal item “I do not need a smear test if I do not have any symptoms” did not significantly differ across any sociodemographic, migration and healthcare groups. Endorsement with “I’m not sexually active so I don’t need to go for a smear test” was found significantly associated with being Asian (6.3%) and African (5.3%), non-employed (3.3%), and never having had a gynecology appointment or the last appointment was 5 or more years ago (4.9%). The statement “I don’t want to know if I have cancer” was most frequently endorsed by women who reported never having had a GP appointment in Portugal (4.1%), and never having had a gynecology appointment or the last appointment was 5 or more years ago (4.0%). The attitudinal item “I intend to go for a smear test, but I don’t get around to it” was more frequently endorsed by women who never had a GP appointment in Portugal (7.4%), and those who do not have a family doctor (4.9%). Concerning “I’ve had a bad experience of a smear test in the past”, significant differences were observed regarding age, country of birth, and having children. Women who agree with the statement were mainly ≥ 45 years old (16.5%), from Europe (17.8%) or North America or Oceania (21.9%), and do not have children (15.2%).

**Table 3:** Characteristics of the participants who endorsed attitudinal barriers associated with CCS non-attendance attendance

	I’m not sexually active so I don’t need to go for a smear test			I do not need a smear test if I do not have any symptoms			I don’t want to know if I have cancer			I’ve had a bad experience of a smear test in the past			I intend to go for a smear test, but I don’t get around to it		
	Agree (%)	Disagree (%)	*p*	Agree (%)	Disagree (%)	p	Agree (%)	Disagree (%)	*p*	Agree (%)	Disagree (%)	*p*	Agree (%)	Disagree (%)	*p*
*Socioeconomic characteristics*															
Age															
< 45 years	2.2	97.8	0.820	1.7	98.3	0.773	2.1	97.9	0.465	16.5	83.5	0.002	3.6	96.4	0.756
≥ 45 years	2.0	98.0		1.5	98.5		2.8	97.2		9.9	90.1		4.0	96.0	
Continent of birth															
Europe	2.3	97.7	0.017	1.4	98.6	0.200	2.9	97.1	0.236	17.8	82.2	< 0.001	3.5	96.5	0.448
Africa	5.3	94.7		4.0	96.0		2.7	97.3		5.4	94.6		2.7	97.3	
Asia	6.3	93.8		4.7	95.3		4.7	95.3		3.1	96.9		0	100	
North America and Oceania	1.4	98.6		1.4	98.6		4.1	95.9		21.9	78.1		5.5	94.5	
Central and South America	0.9	99.1		1.4	98.6		1.2	98.8		7.9	92.1		4.2	95.8	
Education															
Elementary or middle school (≤ 9 years of school)	0	100	0.409	0	100	0.161	5.6	94.4	0.667	0	100	0.230	5.6	94.4	0.874
High school (10–12 years of school)	3.1	96.9		3.1	96.9		2.4	97.6		13.7	86.3		3.9	96.1	
Higher education (university)	1.9	98.1		1.4	98.6		2.3	97.7		12.3	87.7		3.5	96.5	
Employment status															
Employed	1.1	98.9	0.013	2.0	98.0	0.602	2.6	97.4	0.678	11.1	88.9	0.174	4.5	95.5	0.120
Non-employed	3.3	96.7		1.6	98.4		2.2	97.8		13.9	86.1		2.7	97.3	
Marital status															
Married/living with a partner	1.6	98.4	0.068	1.5	98.5	0.276	2.2	97.8	0.530	12.6	87.4	0.689	3.8	96.2	0.756
Single/separated/divorced/widow	3.4	96.6		2.4	97.6		2.8	97.2		11.7	88.3		3.4	96.6	
Having children															
Yes	1.5	98.5	0.100	1.5	98.5	0.441	2.7	97.3	0.400	84.8	89.8	0.014	3.7	96.3	0.936
No	3.0	97.0		2.1	97.9		1.9	98.1		15.2	10.2		3.6	96.4	
*Migration-related characteristics*															
Current migration situation															
Documented	2.0	98.0	0.428	1.5	96.2	0.072	2.3	97.7	0.531	12.6	87.4	0.602	3.3	96.7	0.105
Undocumented	3.1	96.9		3.8	98.5		3.1	96.9		10.9	89.1		6.2	93.8	
Length of stay in Portugal															
≤ 10 years	2.0	98.0	0.367	1.6	98.4	0.379	2.0	98.0	0.114	12.9	87.1	0.259	4.2	95.8	0.071
> 10 years	3.0	97.0		2.5	97.5		4.0	96.0		10.0	90.0		1.5	98.5	
*Health-related characteristics*															
Lifetime GP appointment in Portugal															
Yes	1.9	98.1	0.374	1.6	98.4	0.404	1.8	98.2	0.038	11.9	88.1	0.383	2.6	97.4	< 0.001
No	2.9	97.1		2.5	97.5		4.1	95.9		14.0	86.0		7.4	92.6	
Last gynaecological appointment															
< 5 years	1.1	98.9	< 0.001	1.7	98.3	0.620	1.8	98.2	0.042	12.0	88.0	0.529	3.2	96.8	0.173
≥ 5 years	4.9	95.1		2.1	97.9		4.0	96.0		13.4	86.6		5.0	95.0	
Having a family doctor in Portugal															
Yes	2.6	97.4	0.281	1.6	98.4	0.551	2.3	97.7	0.833	12.0	88.0	0.672	2.6	97.4	0.048
No	1.6	98.4		2.0	98.0		2.5	97.5		12.9	87.1		4.9	95.1	
HPV vaccine															
Yes	1.2	98.8	0.387	0.6	99.4	0.339	0.6	99.4	0.114	8.1	91.9	0.072	1.9	98.1	0.188
No	2.3	97.7		2.0	98.0		2.7	97.3		13.1	86.9		4.0	96.0	

## Discussion

The findings of this study show that fear of the test result, worry about seeing a male health professional, perceiving the test as painful, embarrassment, difficulties scheduling the test, and having a negative experience in screening are the most reported attitudinal barriers among migrant women, with an agreement percentage ranging from 12.4 to 25.3%. On the other hand, the lowest percentage of agreement were observed in the items of “perceived need of screening” (agreement percentage of 6.6% or below). In contrast, CCS non-attendance was found significantly associated with low perceived need in the absence of symptoms and lack of motivation to be screened.

Overall, it was observed a non-attendance proportion of 24.4% among the migrant women included. This percentage is lower than what has been observed in other studies conducted in Europe (50–56%) [18, 20, 29–31], which may be explained by the differences in attendance definition used in studies, but also in the socio-characteristics of the studies populations.

Attitudinal barriers may have impact on CCS non-attendance [22, 23, 25–27, 32]. In our study, the most often endorsed attitudinal barriers were related to “concerns about the test”, similarly to what Marlow et al. [16] found in the study conducted in England. However, the percentages are lower in the present study. For instance, the percentage of women stating that screening was embarrassing was 18.5% which is considerably lower than the 59.1% reported for Black, Asian, and Minority Ethnic women in England. However, contrary to the Marlow et al. study [16], endorsement of items related to the “perceived need for screening” was low. The discrepancies in findings may be due to differences in characteristics of the samples, such as the countries of birth. The English study included women from the Caribbean region, Africa, Pakistan, India, and Bangladesh, while this study sample was mostly from Europe and South America. Alternative explanations for discrepancies between the studies’ findings are the higher average educational level and the lower rate of CCS non-attendance in our study sample. Overall, this study highlights the importance of exploring the attitudinal barriers described by Marlow et al. in different migrant populations and geographic contexts as they may differ across cultural backgrounds and host countries.

Despite the higher endorsement of these factors among participants, neither of them seems to stop these women from attending CCS, as no significant associations were found between these items and CCS non-attendance. However, some studies point out embarrassment, pain, negative past experiences and the role of male healthcare professionals as barriers to CCS [6, 7, 32]. It is important to note that these results suggest a dissociation between “feeling the barrier or agreeing with it” and “actual influence of the barrier in preventing screening”. The reasoning why a woman decides whether to attend CCS is complex. Beliefs, perceptions, knowledge, and cultural values also take part on how a woman perceives the need to attend CCS. Johnson et al. [6] states that cultural background plays a role on women’s perceptions and attitudes towards CCS, and it can vary with their geographic location and country of birth. In the present study, a large portion of migrant women came from countries with established CCS programs (e.g., Brazil and several Western European Countries). Possibly, women from these countries may be more aware and open to preventive health measures and, therefore, they attend the test regardless of how they feel about the procedure [18–20].

Overall, even though our findings suggest that the attitudinal barriers associated with CCS non-attendance are mainly related to “perceived need of screening”, other attitudinal barriers, such as “fear of cancer” and “practical considerations”, were also stated. Particularly, the idea of low perceived need of screening, lack of interest in knowing if they have cancer and not prioritizing doing the screening were associated with CCS non-attendance. The same trend is observed elsewhere [6, 7, 32], including in the Marlow et al. [16] study. Other factors, such as the way women perceive their own health and health needs, including the need of doing the test, and the fear of feeling discriminated because of their cultural or physical characteristics (e.g. high BMI) may influence CCS non-attendance [7, 18, 21, 33]. These results suggest that future interventions to increase women’s awareness of the benefits of CCS and its importance could contribute to promote their participation.

In this study, migrant women who reported having had a bad experience in CCS in the past were less likely to be CCS non-attenders. This seems contrary to evidence suggesting that negative experiences during screening are more likely to increase non-attendance among women [7, 24, 34, 35]. Considering that this is a cross-sectional study, this finding may be explained by reverse causality. Women who had a bad experience in CCS necessarily have done the screening at least once in their lifetime. The fact that, even so, these women report being CCS attenders may be due to two reasons: (1) the bad experience in CCS they had in the past occurred less than 5 years ago (meaning that they are still within the normal screening interval and not considered non-attenders), and (2) the bad experience these women had was not enough reason to stop them from doing the screening. It would be important to explore why women considered their experiences negative to better improve the quality of CCS delivered to them. To further understand who the non-attenders are, an additional characterization of the women who endorsed the attitudinal barriers associated with CCS non-attendance was performed. Women who reported having had a negative experience in screening were younger, from high income countries and did not have children. To have a negative experience, a woman needs to have been through screening, and therefore reverse causality may occur in this situation. Also, evidence shows that these socioeconomic characteristics may be facilitators to CCS as described elsewhere [7, 17]. This may explain why endorsement of this statement was found to be inversely associated with CCS non-attendance.

Lack of regular contact with healthcare services (GP and/or Gynaecologist) seems to have a negative effect on CCS attendance. This highlights the importance of having a close relationship with a medical doctor, who not only provides medical care but also informs about preventive measures and helps improve health literacy [6, 7, 17].

This study’s findings may be useful for policy makers and stakeholders’ practice. By quantifying which barriers are associated with CCS non-attendance, this study provides insights of which areas should be targeted to increase participation in screening among these groups. On the other hand, there are also barriers about which women agree with (e.g., pain and embarrassment, seeing a male doctor) that do not seem to influence CCS attendance and therefore may be of lower priority when creating an intervention with limited resources. This study provides insights for potential interventions to increase screening participation in migrant women. Overall, having regular contact with a trusting healthcare provider seems to be a key factor for CCS participation. The misconception that “I do not need a smear test if I do not have any symptoms” may be addressed by individual counselling by clinicians, by targeted health campaigns, or both. Not prioritizing screening (“I intend to go for a smear test, but I don’t get around to it”) may require combination of education, positive peer modelling, and enablement (i.e. increase opportunity for screening) interventions. These educational interventions could also be performed in partnership with community workers that may help providing culturally and linguistically adapted information, increasing the effectiveness of the strategies [23, 36]. These strategies may increase health literacy among migrant women and provide them with knowledge to make informed decisions regarding cervical cancer screening. Finally, self-sampling screening tests as an alternative to conventional screening may be an effective strategy to reduce barriers to screening, namely in terms of shame, fear of the test, or lack of time, allowing women to perform the test themselves in a place of their choice [23, 37–39].

### Strengths and limitations

This study includes a large and diverse sample. The use of an online survey helped to reach a high number of participants, particularly during the COVID-19 pandemic outbreak. This online data collection method reduces human error on data insertion as this process is made automatically in the survey platform. The use of measures previously used in another country (attitudinal barriers statements) allows to compare results between studies.

This study has some limitations that should be considered. This is a cross-sectional study; hence it is not possible to assess the causality between variables. Also, being a web-based survey, only women with access to the internet could participate, which can lead to sample bias. The online nature of the survey and the fact that the survey was provided only in Portuguese and English may also justify the high percentage of women with higher education in the sample. The results of the study cannot be generalized to the whole migrant population in Portugal because, even though the sample is large and diverse, it is not representative of the migrant population. Women aged 20–24 years old (under the age limit of the screening program in Portugal) were included in the study, to analyze as many perspectives as possible, including those of young women who will be eligible for population-based CCS in the near future. In a sensitivity analysis excluding these women a similar distribution of participants’ characteristics was found and no changes in the results of the logistic regression analysis were observed.

## Conclusions

Attitudinal barriers play a role on CCS attendance among migrant women. Factors such as fear, embarrassment, or even modesty associated with seeing a male doctor have been reported by several women. However, experiencing an attitudinal barrier does not necessarily lead women to decide not to screen. This is clear if considering that the most reported barriers in this study are not associated with CCS non-attendance. CCS non-attendance was associated with lower perceived need of screening in the absence of symptoms and lack of motivation to attend screening. By understanding what are the barriers that seem to compromise the CCS attendance, stakeholders may use that information to develop interventions addressing those sensitive areas. This offers a window of opportunity to work these potential barriers a priori, developing more effective strategies aiming to increase CCS attendance among migrant women. Strategies and interventions aiming to increase CCS participation among migrant women should target these barriers. Strategies aiming to increase health literacy and empower female migrants may be key to increase screening attendance among these populations.

## Data Availability

The dataset generated during the current study are available from the corresponding author on reasonable request.

## Abbreviations

CCS: Cervical cancer screening
GP: General practitioner
